# High Prevalence of Dysfunctional Animal–Visitor Interactions in 225 Southeast Asian Zoos and Aquariums

**DOI:** 10.3390/ani14223167

**Published:** 2024-11-05

**Authors:** Anna Fourage, Tanya Erzinclioglu, Amanda Fairey, Marco Campera, K. Anne-Isola Nekaris, Vincent Nijman

**Affiliations:** 1 School of Law and Social Sciences, Oxford Brookes University, Oxford OX3 0BP, UK; fourageanna@gmail.com (A.F.); amfairey@gmail.com (A.F.); anekaris@littlefireface.org (K.A.-I.N.); 2 For Tigers, Cambridge CB23 1HB, UK; tanya@fortigers.org; 3 Department of Biological and Medical Sciences, Oxford Brookes University, Oxford OX3 0BP, UK; mcampera@brookes.ac.uk; 4 School of Life Sciences, Anglia Ruskin University, Cambridge CB1 1PT, UK

**Keywords:** accreditation, animal welfare, conservation, education, entertainment, zoological associations

## Abstract

Humans have long delighted in being entertained by captive wild animals. In zoos and aquariums, animal–visitor interactions allow visitors to experience direct or indirect contact with animals. Well-managed animal–visitor interactions can help zoos and aquariums contribute to their educational objectives by eliciting pro-conservation behaviours from visitors while providing good animal welfare for the animals involved. However, some interactions are dysfunctional because they cause physical and psychological harm to the animal and reinforce negative values and beliefs towards conservation. We aimed to assess how dysfunctional animal–visitor interactions are and whether accreditation by zoological associations reduced the number of such interactions. We visited Thai zoos in person and conducted online monitoring of facilities in Southeast Asia. We report 1165 animal–visitor interactions in nine Southeast Asian countries in 225 zoos and aquariums. Taking photos with animals (“selfies”) and riding on animals were scored as the animal–visitor interactions that were the most dysfunctional. The number of animal–visitor interactions was not less in accredited zoos, and there was little difference among countries. Behaviour change needs to form a strategy for change, in addition to robust legislation and improved efforts by zoological associations to push members to maintain standards and mentor non-accredited facilities.

## Simple Summary

Animal–visitor interactions are a component of zoo and aquarium entertainment and include activities such as petting, taking selfies, feeding, and animal shows and presentations. Well-managed animal–visitor interactions should provide good animal welfare and promote pro-conservation behaviours in visitors. In reality, some animal–visitor interactions may be dysfunctional as they create more harm than good, which undermines some of the objectives of a modern zoo and goes against zoological associations’ guidelines on animal–visitor interactions. We focused our study on Thai zoos, where we surveyed animal–visitor interactions in person and assessed the dysfunctionality of interactions based on animal welfare and conservation education messaging. In addition, we conducted an online search for animal–visitor interactions at 225 Southeast Asian zoos and aquariums, recording the type, prevalence, species used, and whether this was impacted by accreditation by a zoological association. We found photos (“selfies”) and riding interactions most dysfunctional and that a high proportion of facilities surveyed in Southeast Asia offer dysfunctional animal–visitor interactions. Stopping these types of interactions is challenging without implementing and enforcing robust legislation concerning the use of animals in entertainment and changing the attitudes and behaviour of zoo and aquarium visitors through education.

## 1. Introduction

The desire of humans to be close to animals and entertained by them dates back at least to 4500 BC and the early menageries of the powerful and wealthy [1,2,3]. Since the 1960s, zoos have faced significant pressure to position themselves as centres of conservation rather than simply places of entertainment [4]. In part, this was to improve their social license, following criticism of a lack of contribution to conservation and condemnation from animal rights organizations that zoos should not exist at all [5,6,7,8]. The stark reality remains that a majority of people visit zoos primarily for recreational purposes and overwhelmingly expect to be entertained [7,9,10,11,12,13], although some visitors also anticipate an educational component to their visit [9,14]. Still, the fact is that zoos and aquariums heavily rely on visitors to finance their operations [6,12]. Therefore, conflicts may arise between fulfilling the expected roles of a modern zoo and aquarium, i.e., positive inputs to conservation, research, education, animal welfare, and entertainment (with educational components) [8,15] and the need to attract enough visitors by providing sufficient entertainment to achieve profitability [11,16].

One form of visitor entertainment is animal–visitor interactions, defined as interactions that allow visitors to have direct or indirect contact with animals through activities such as hand-feeding, petting, or watching shows and performances [17]. Animal–visitor interactions can be broadly assessed to determine whether they have positive, negative, or neutral benefits to biodiversity conservation and animal welfare, similar to the way Moorhouse et al. [18] classified the impact of different types of wildlife tourist attractions. Well-managed animal–visitor interactions can assist zoos’ and aquariums’ educational goals in encouraging pro-conservation behaviours in visitors as they can learn more about the importance of conserving species in their natural habitats, consequently fostering greater respect for animals in the wild [19]. Equally, as suggested in Wild Welfare’s Position Statement on Animal-Visitor Interactions [20], some animal–visitor interactions may be beneficial to animal welfare in the form of enrichment. Yet, the literature on how animal–visitor interactions positively impact welfare is still sparse [17], with data limited to the influence of visitor effect on a few species, such as primates [11,21,22,23], bears [24], and giraffes [25].

Walker [26] introduced the concept of a dysfunctional zoo “to describe a type of captive wild animal facility that does not function adequately (or at all) for even the most essential canons of zoos, e.g., education, conservation or research”. We narrowed this concept down to “dysfunctional animal–visitor interactions” within zoos and aquariums. Firstly, these interactions cause more harm than good and, secondly, as with Walker’s dysfunctional zoos, they do not agree with the most essential canons of zoos.

There are numerous damaging consequences of dysfunctional animal–visitor interactions [15,27,28,29,30]. For example, inappropriate interactions with captive wildlife can harm conservation because it normalizes close contact with wild animals [17,31] and undermines educational messaging concerned with protecting species and their habitats [32]. Furthermore, dysfunctional animal–visitor interactions do not promote pro-conservation behaviours, as visitors generally have poor judgment on which interactions are detrimental [18,29]. Images of humans interacting with wild animals in the media were found to stimulate the demand for participation in animal–visitor interactions [33] and increase people’s desire to own an exotic pet [34,35,36]. There is also the abundant literature detailing how the forced proximity of animal–visitor interactions can negatively impact welfare, such as inducing stress and unnatural behaviours, including in many of the different types of wildlife tourist attractions, as assessed by Moorhouse et al. [18].

The World Association of Zoos and Aquariums (WAZA) currently accredits some 1300 facilities through 21 regional zoological associations and some affiliate members [37]. Yet, globally, most facilities remain unaccredited. Associations such as WAZA provide guidelines to members on providing animal–visitor interactions. For example, the zoo association within the region of our study’s focus, the Southeast Asian Zoo Association (SEAZA), stipulates that “SEAZA believes animal demonstrations and animal-visitor interactions that are detrimental to the physical or psychological wellbeing of the animals are unacceptable. These include circus-like shows, demonstrations, animal photography, or interactions that are not respectful (for example, showing an animal in funny clothing), demonstrate unnatural behaviours, and are detrimental to an animal’s immediate and long-term physical and psychological wellbeing” [38]. WAZA also has a similar set of guidelines [39]. The adherence to guidelines is dependent on the individual zoo and aquarium and may even be wholly disregarded [29]. Therefore, even accredited zoos and aquariums may offer dysfunctional animal–visitor interactions, potentially impacting thousands of animals subjected to stressful conditions for visitor entertainment and profit. A global review by D’Cruze et al. [17] provided a constructive audit of the diversity of animal–visitor interactions in zoos and aquariums worldwide. Their study remains the only comprehensive data on the types and occurrence of animal–visitor interactions in zoos. However, D’Cruze et al.’s study [17] only included facilities accredited either through direct membership with WAZA or through WAZA-affiliated regional or national zoo associations, meaning that the prevalence of animal–visitor interactions in non-accredited facilities remains unknown. Non-accredited facilities are likely to have more dysfunctional animal–visitor interactions due to a total lack of oversight and are an important sector of zoos and aquariums to evaluate to determine the true extent of the animal–visitor interaction issue.

Southeast Asia is a region comprising 11 countries, an estimated human population of 696.5 million [40], and a predicted economic growth rate of 4.7% for 2025 [41]. It has a wide range of zoos and wildlife tourist attractions. Within Southeast Asian countries, there are varying degrees of legislation on animal welfare, zoo standards, and regulations on the use of animals in entertainment [42,43]. The presence of animal–visitor interactions within Southeast Asian zoos and aquariums is still largely undocumented, except for some reports that primarily focus on Thailand and a handful of species, including tigers (*Panthera tigris*) [42,44], Asian elephants (*Elephas maximus*) [42,43], and apes [45,46,47].

To date, there has not been an audit of the way in which captive wildlife in zoos and aquariums are utilized for entertainment in Southeast Asia. Therefore, we aimed to conduct a review, similar to D’Cruze et al. [17], to (1) provide an analysis of animal–visitor interactions in Thai zoos and assess how dysfunctional they are; (2) document the number and type of animal–visitor interactions in Southeast Asian zoos and aquariums; (3) identify the species class of animals used in animal–visitor interactions; and (4) evaluate whether accreditation status impacts the animal–visitor interactions offered. Our study aimed to highlight how widespread dysfunctional animal–visitor interactions are in the region. As such, we hoped to illustrate the urgent need for improved legislation and regulation on zoo standards and animals used in entertainment. Furthermore, we intended to call attention to the greater efforts required by zoological associations to advance standards and compliance within their own member institutions. We emphasized the need for accredited zoos and associations to work with non-accredited facilities to improve standards and eliminate all animal–visitor interactions detrimental to conservation and welfare.

## 2. Materials and Methods

### 2.1. Study Region and Definitions

We included the following Southeast Asian countries: Brunei, Cambodia, Indonesia, Laos, Myanmar, Malaysia, the Philippines, Singapore, Thailand, Timor-Leste, and Vietnam. In the last decade, the authors conducted zoo assessments in seven of the 11 Southeast Asian countries and collectively visited 112 facilities in the region. We searched for zoos and aquariums in each country by searching Google directly and ZooChat and by checking the lists published on national zoo associations and the SEAZA websites. Once we identified the facility in a country, from June to October 2024, one researcher systematically searched multiple websites, including the zoo website, Facebook, Instagram, Google Reviews, and TripAdvisor, for animal–visitor interactions. One researcher visited all the zoos, but not the aquariums, in Thailand at least twice between July 2020 and October 2024. Being present onsite to witness the animal–visitor interaction enabled us to record the animals used at the species level. By using the same criteria as [17] we made our study comparable to previous studies (Table 1).

We defined a zoo and/or aquarium as a facility open to the public that displays multiple species of captive wildlife. We excluded facilities holding single species of wild animals, such as elephant camps, tiger parks, and facilities only offering monkey shows, crocodile shows, and snake shows, despite these establishments being abundant in the region. We also excluded facilities such as butterfly farms, petting zoos for domesticated species (we excluded all domesticated animals from this study, including camelids), and sanctuaries. For facilities with “sanctuary” in the name, we double-checked whether the facility was a sanctuary as defined by the Global Federation of Animal Sanctuaries (GFAS). They define a sanctuary as “any facility providing temporary or permanent safe haven to animals in need while meeting the principles of true sanctuaries: providing excellent and humane care for their animals in a non-exploitative environment and having ethical policies in place” [48].

### 2.2. Data Collection

We developed a scoring framework comprising different criteria that can impact the welfare of animals during animal–visitor interactions that we used in Thai zoos only. We considered Mellor et al.’s Five Domain model, updated to include human–animal interactions [49], when developing the framework. In addition, we added a measure to quantify the value of conservation messaging (Table 2). The researcher also took notes on the interactions to provide a qualitative description of what the animal–visitor interaction type entailed.

We recorded all animal–visitor interactions in zoos and aquariums found online and visited in person. We followed the definition of animal–visitor interactions used by D’Cruze et al. [17] and included an additional animal–visitor interaction type, “photo” (Table 1). There were instances where an animal–visitor interaction could potentially fulfil the criteria for multiple animal–visitor interaction types. For instance, an animal–visitor interaction involving a visitor having their photo taken whilst feeding a tiger cub with a bottle of milk was classified as feeding if the interaction primarily advertised feeding over the photo. If we did not observe any animal–visitor interactions in a zoo, we recorded that no interactions were observed. We checked the WAZA and SEAZA websites to see if the facility was accredited.

### 2.3. Data Analysis

Data were cleaned to ensure all domestic species were removed and all facilities met the criteria for inclusion in the study. A second author double-checked the entries for accuracy and completeness, and, if a discrepancy was detected, an agreement was reached through consultation. For the focus on Thai zoos, we excluded 58 of the 339 animal–visitor interactions from the analysis for Order and families as these 58 interactions involved many families too numerous to analyse properly, for example, in walk-through aviaries with many different bird species or hand-feeding in a zoo whereby visitors could freely feed any animal. We therefore provided a breakdown of species listed at the family level in 281 animal–visitor interactions. For the dysfunctionality score in Thai zoos, we ran a Spearman test via ‘ggscatterstats’ function for R 4.3.1 to determine the correlation coefficients (ρ) between the dysfunctionality score and animal–visitor interactions. We considered 34 zoos in Thailand for which we had this score. The analysis is based on the level of species class. When the animal–visitor interaction involved animals of two or more classes, we recorded all of them for that animal–visitor interaction.

We compared the proportion of facilities offering animal–visitor interactions among countries and the proportion of accredited versus non-accredited facilities, and we compared the different species classes of interactions. For countries with four or more zoos, we tested if more or fewer were not accredited than what would be expected if accreditation levels were fully equal in all of Southeast Asia, using chi-square tests using Yates’ corrections for continuity. We generated expected values under the assumption that 17% of all zoos in our study were indeed accredited. We tested if accredited zoos (all countries combined) had a different prevalence of certain animal–visitor interactions than non-accredited zoos (again, all countries combined) by means of testing for homogeneous distributions using chi-square tests using Yates’ corrections. Expected values were generated on the basis of the number of accredited vs. non-accredited zoos. We then repeated this, focusing on just zoos in Thailand. We accepted significance when *p* < 0.05 in a two-tailed test, although, occasionally, we mentioned trends when *p* < 0.10.

## 3. Results

### 3.1. Animal–Visitor Interactions in Thai Zoos

**Figure 1:**
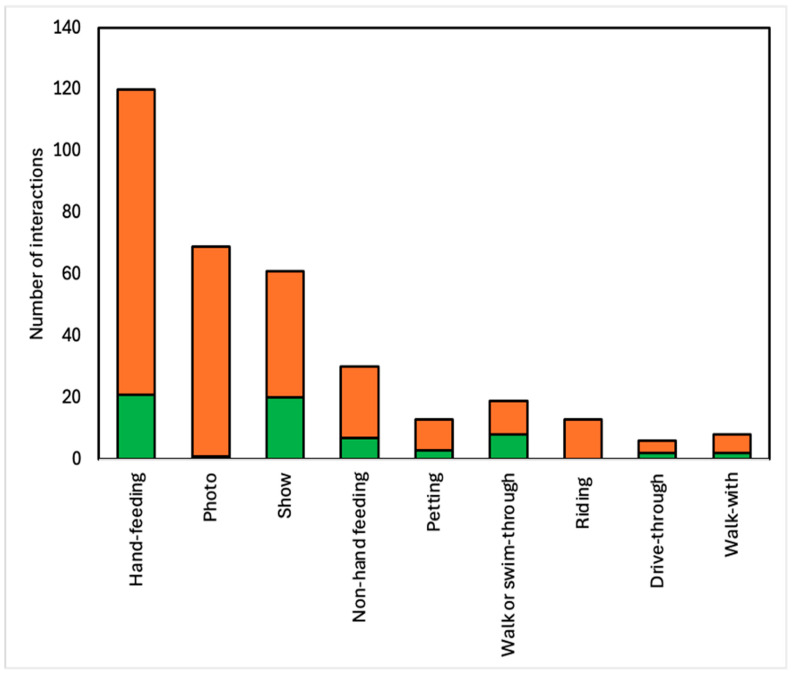
A total of 339 animal–visitor interactions by type and zoo accreditation status in Thai zoos. Green represents interactions in accredited zoos, and orange represents interactions in non-accredited zoos.

We found that 96% (*n* = 53) of the 55 Thai zoos we visited in person offered 339 animal–visitor interactions, with the eight accredited zoos having 64 animal–visitor interactions (mean = 8) and the 45 non-accredited zoos having 275 animal–visitor interactions (mean = 275). Hand-feeding was the most common animal–visitor interaction type in both accredited and non-accredited zoos. The least common interaction type in accredited zoos was riding, with zero interactions, and photo, with one interaction. In non-accredited zoos, the least common animal–visitor interaction was drive-through (*n* = 4) (Figure 1).

**Table 3:** Qualitative description of the different types of animal–visitor interactions as observed in Thai zoos.

Animal–Visitor Interaction	Observations
Petting	Petting animal–visitor interactions were uncommon as most petting interactions involved domestic animals such as rabbits, goats, sheep, and pigs (excluded in this study). Generally, the petting of captive wild animals involved reptiles, particularly pythons (Figure 2A). One zoo offered a petting experience with a fennec fox (*Vulpes zerda*), where visitors sat with the animal on their lap. Another zoo allowed visitors to pet juvenile crocodiles. The crocodiles were kept in tanks, and their snouts were taped closed. The visitor then held the crocodile in their arms (Figure 2B).
Photo	Zoos advertised animal–visitor interactions, where visitors usually had to pay an additional amount. There were photo opportunities with a wide range of species, including gibbons (Figure 2C). Orangutan photos were also popular, where the animal would usually be dressed in human clothing and sit on the visitor’s lap. The handler would then guide the animal to perform various poses, including kissing the cheeks of the visitor and wrapping an arm around them. Big cat photo opportunities were the most prevalent, where the cat was typically chained by the neck and kept on a small platform (Figure 2D). The handler would guide the visitor to sit in a particular position next to the cat. Most visitors would be physically touching the animal. To provide a better photo, the handler would often prod the side of the cat’s mouth with a stick to make the animal roar. Despite being a nocturnal species, we also observed Siberian eagle owls (*Bubo bubo*) being used as photo props in full sunlight (Figure 2E).
Hand-feeding	Hand-feeding was by far the most common animal–visitor interaction for all zoo types. Food was often presented to visitors with a stand outside a particular exhibit that sold food for the animals in that exhibit. Some hand-feeding entailed visitors being sold a basket of food at the entrance or other points within the zoo, allowing unrestricted feeding for all animals. Here, visitors could feed whichever animals they chose, even if the food was inappropriate for the species. Food would typically include carrots, bananas, and sunflower seeds. Some zoos had very thin animals but sold animal food to visitors. Animals were reportedly not fed before visitors arrived to make animals hungrier so that they would beg for food from visitors. The most common species to hand-feed were big cat cubs (Figure 2F), deer, giraffes, zebras, and capybara (*Hydrochoerus hydrochaeris*).
Non-hand-feeding	Non-hand-feeding animal–visitor interactions needed indirect contact because most species involved were dangerous. In these interactions, visitors were given a long stick with meat on the end and then put the stick through the fence. Crocodile feeding was offered in numerous facilities. Visitors were given a fishing rod with meat attached to the end for crocodile feeding (Figure 3A). Visitors would lower the stick into the crocodile pond and usually move the stick up, down, and over various crocodiles’ heads to elicit a response. Most of the time, the crocodiles would be teased, and visitors would wonder which crocodile would make the move and jump to acquire the meat. Many non-hand-feeding interactions also occurred with big cats such as tigers or lions (Figure 3B).
Riding	All riding animal–visitor interactions with captive wild animals involved elephants. Although excluded from the analysis, we observed facilities offering camel rides and one facility offering domestic water buffalo (*Bubalus bubalis*) rides. Elephants used for riding wore a howdah (a wooden riding seat) on their backs, even when waiting for visitors to pay to ride. They were almost always shackled at the foot while waiting for visitors (Figure 3C).
Walk-through	The majority of walk-through animal–visitor interactions were walk-through aviaries. Some of these exhibits were very large and held many different bird species. Visitors in these aviaries would not expect to have any direct contact with birds. Instead, they expected to be able to view them from afar and perhaps watch them congregate at feeding places within the aviary.
Walk-with	All walk-with animal–visitor interactions that we observed were with big cats. Visitors were ushered into the animal’s enclosure and given the cat’s leash (Figure 2D). Visitors then walked around the enclosure accompanied by a staff member (usually holding a sharp stick), who would occasionally prod the cat to make it cooperate. When not walking, the cats would usually be tethered by a chain.
Drive-through	Only three zoos provided drive-through animal–visitor interactions whereby visitors traveled by bus or tram through a safari-style park with multiple exhibits spread over a large area. In two of these zoos, visitors could drive their cars. Buses and trams had guides onboard who used microphones, which were usually very loud and could be heard far away from the vehicle. The other drive-through animal–visitor interactions were small trams or jeeps that drove through enclosures (giraffes and zebras in two exhibits), and the third drive-through interaction allowed visitors to drive through a large area containing multiple deer species.
Show and performances	Shows varied in species’ composition and content. The animal presentations generally showcased animals’ natural behaviors, such as flying to targets, foraging for food, swimming, and jumping. On the other hand, many shows had animals performing unnatural behaviors or being subjected to cruel treatment by their handlers. For example, crocodiles in shows were routinely hit with a stick and dragged by their tails (Figure 3E). Snakes in shows were also subjected to rough handling and provocation by handlers. Elephants in shows were forced to perform behaviors such as hula hooping, playing football, basketball, and darts. Many were forced to walk bipedally, with their front legs on the back of another elephant. We noticed a sexualization of some shows, including orangutans and elephants “twerking”, defined as “sexually suggestive dancing characterized by rapid, repeated hip thrusts and shaking of the buttocks, especially while squatting” [50] (Figure 3F).

**Figure 2:**
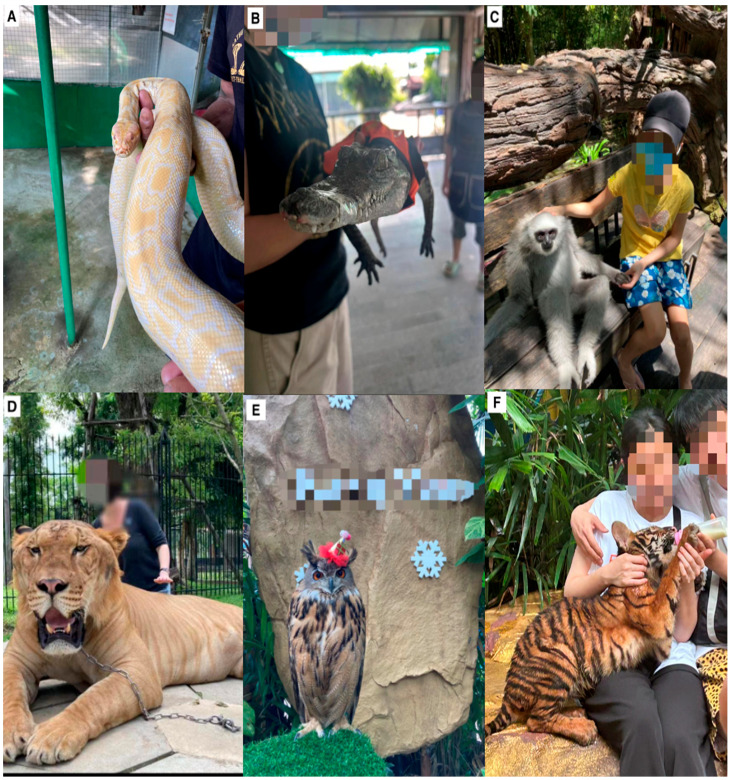
Photos of animal–visitor interactions in Thai zoos. (**A**) Python in a petting interaction; (**B**) crocodile in a petting interaction with its snout taped shut and wearing clothes; (**C**) young girl with gibbon in a photo interaction; (**D**) liger (lion–tiger hybrid) in a photo animal–visitor interaction; (**E**) owl on standby for a photo interaction; (**F**) a tiger cub being bottle-fed. We saw that the cub was placed on a visitor’s lap for 20 s before being placed on another visitor’s lap to resume feeding. Accounting for the time for the new visitor to sit down, this meant that the cub was placed on 20 different visitors’ laps in the space of 10 min. Photos were all taken by the first author.

**Figure 3:**
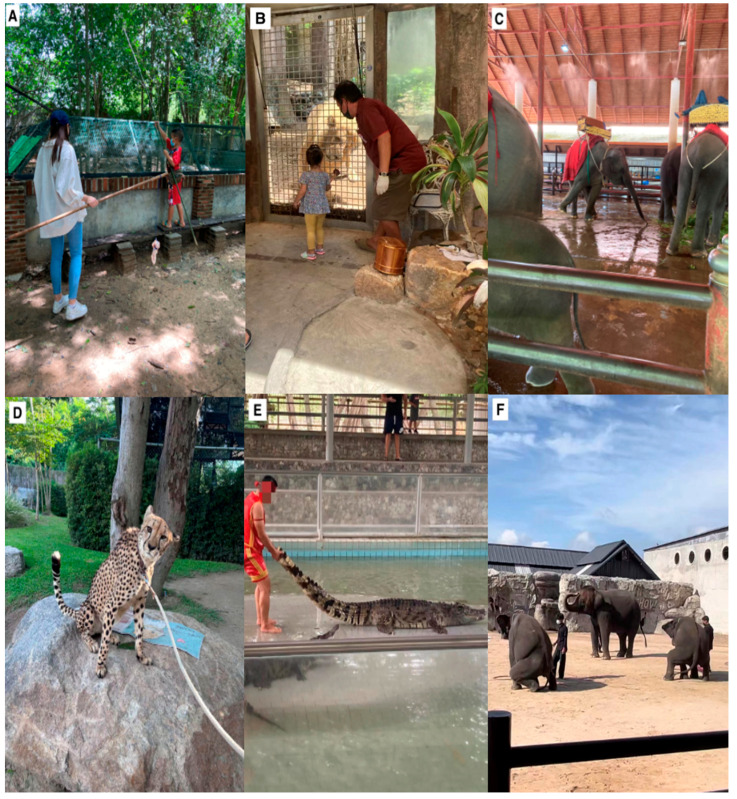
(**A**) Non-hand-feeding crocodiles with chicken attached to a “fishing rod”. The crocodiles in the pit below are usually teased, with the person holding the stick jerking it up and down to try and make the crocodiles jump; (**B**) young girl feeding a lion through bars in a non-hand-feeding interaction; (**C**) elephant wearing a howdah and shackled at the ankle trying to move; (**D**) cheetah wearing a leash about to go on a walk-with animal–visitor interaction; (**E**) a crocodile having its tail pulled by a handler in a crocodile show; (**F**) elephants “twerking” in an elephant show. Photos were all taken by the first author.

Twenty of the 61 shows were in accredited zoos. Nineteen shows had educational components, of which 12 were animal presentations, showcasing animals performing species-specific behaviours, such as foraging, stalking, climbing, and flying. Six of these presentations featured mixed species, usually including hornbills, binturong (*Arcticus binturong*), and Asiatic small-clawed otters (*Aonyx cinereus*). There were also six tiger enrichment presentations, whereby a feeding line was baited with meat in the tiger enclosure, and visitors could watch the tiger jump/climb up a pole to retrieve the meat. One dysfunctional show in an accredited zoo featured tigers and lions performing unnatural behaviours, including walking bipedally, rolling a barrel, sitting on hind legs in a squat position, and rolling over. The 41 shows in the non-accredited zoos were all dysfunctional, except for three bird presentations that showcased natural behaviours. Crocodile shows were the most common (*n* = 10), followed by elephant shows (*n* = 8). Primate shows (*n* = 7) involved both long-tailed macaques (*Macaca fascicularis*) and northern pig-tailed macaques (*M. leonina*) in four shows and orangutans in three shows. Snake shows (*n* = 6) predominantly featured a mix of King cobra (*Ophiophagus hannah*) and Burmese pythons (*Python bivittatus*). Finally, we observed tiger/lion shows (*n* = 5). We also observed various activities for each animal–visitor interaction type illustrated in Figure 2 and Figure 3 and described in Table 3.

**Figure 4:**
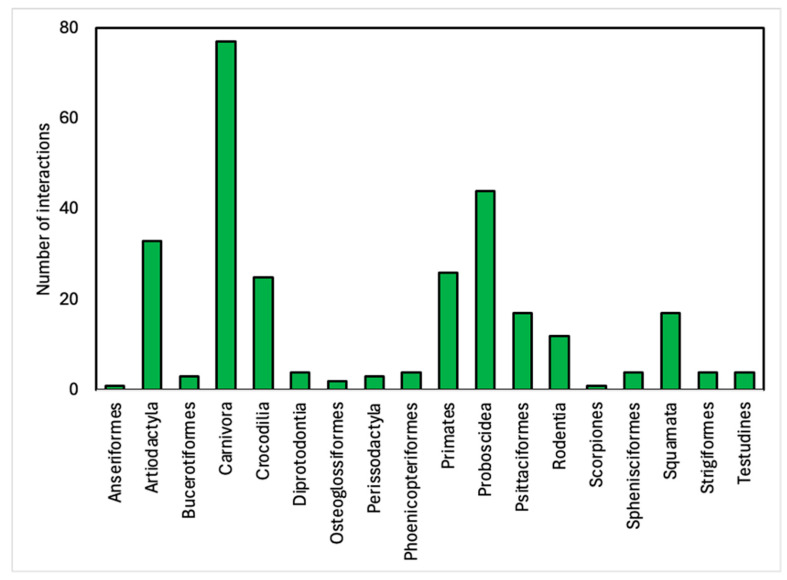
Summary of Orders of species used in 281 animal–visitor interactions in Thai zoos.

**Figure 5:**
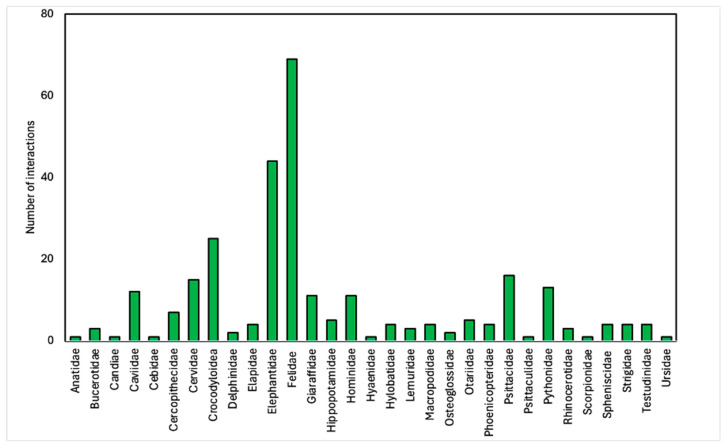
Breakdown of the families of species used in 281 animal–visitor interactions in Thai zoos.

Carnivora was the most common of the 281 animal–visitor interactions we analysed at the Order level (Figure 4). At the Family level, at a minimum, 31 different Families were represented (Figure 5). We could see a clear preference for the use of big cats for animal–visitor interactions. Felidae represented the largest family of species, with almost 25% (*n* = 69) of total interactions. Big cats were included in photo animal–visitor interactions (n = 26), hand-feeding (primarily bottle-feeding of cubs) (*n* = 15), shows (*n* = 12), non-hand-feeding (*n* = 10), and walk-with interactions (*n* = 6). Proboscidea (elephants) were the second largest family group represented due to the high number of animal–visitor interactions involving this species in photos, hand-feeding, riding, and shows.

### 3.2. Dysfunctionality Score of Animal–Visitor Interactions in Thai Zoos

**Figure 6:**
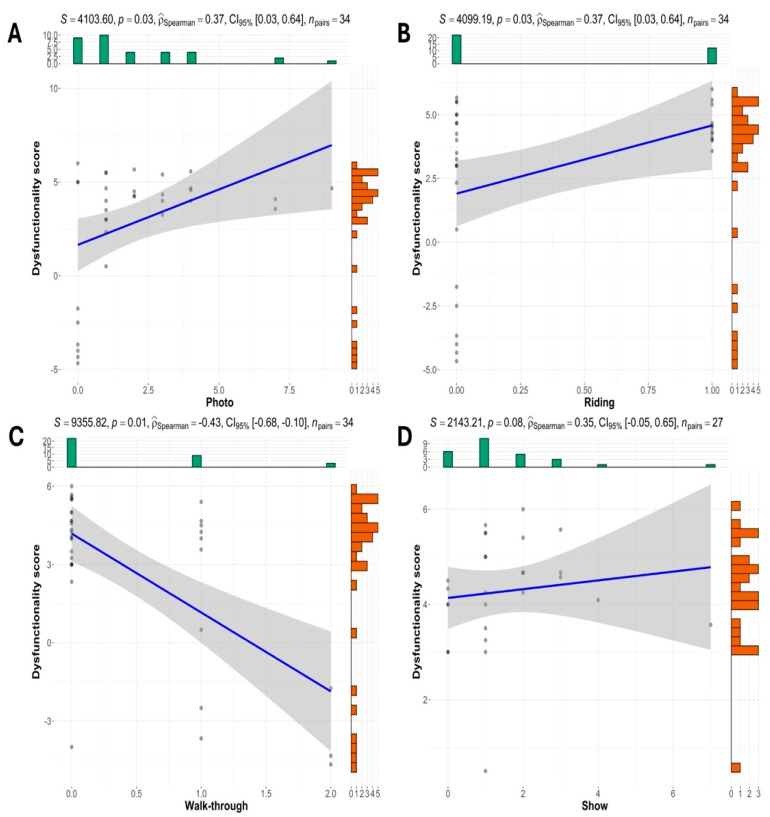
Significant correlations between dysfunctionality score and photo (**A**), riding (**B**), or walk-though (**C**) considering 34 zoos in Thailand, and tendency towards a significant positive correlation between dysfunctionality score and show (**D**) only considering the 27 non-accredited zoos.

The dysfunctionality score was not significantly correlated with hand-feeding (ρ = 0.13, 95%CI: −0.23, 0.45; *p* = 0.48), non-hand-feeding (ρ = 0.00, 95%CI: −0.35, 0.35, *p* = 1.00), show (ρ = −0.17, 95%CI = −0.49, 0.19; *p* = 0.34), petting (ρ = −0.17, 95%CI: −0.48, 0.19, *p* = 0.35), drive-through (ρ = −0.06, 95%CI: −0.40, 0.29, *p* = 0.72), walk-with (ρ = 0.05, 95%CI: −0.30, 0.39, *p* = 0.79), or the total AVIs (ρ = −0.03, 95%CI: −0.37, 0.32, *p* = 0.88). The dysfunctionality score was positively correlated with photo (ρ = 0.37, 95%CI: 0.03, 0.64, *p* = 0.03) and riding (ρ = 0.37, 95%CI: 0.03, 0.64, *p* = 0.03) and negatively correlated with walk-through (ρ = −0.43, 95%CI: −0.68, −0.10, *p* = 0.01) (Figure 6). When only considering non-accredited zoos (n = 27), there was a tendency towards a positive correlation between dysfunctionality score and show (ρ = 0.35, 95%CI: −0.05, 0.65, *p* = 0.08).

### 3.3. Proportion of Animal–Visitor Interaction Types in Southeast Asian Zoos

We identified 255 facilities (219 zoos and 36 aquariums) in 10 Southeast Asian countries (no facilities were found in Timor-Leste). Of these 255 facilities, 44 (17%) were accredited by SEAZA and 211 (83%) were non-accredited. No facilities were members of WAZA only; instead, all were WAZA affiliated through their respective accreditation with SEAZA. Out of the 255 facilities, 225 (88%) had animal–visitor interactions, with no evidence in 30 facilities (12%) of animal–visitor interactions being offered (Table 4). No animal–visitor interactions were observed in the two zoos in Laos P.D.R. In total, we identified 1165 animal–visitor interactions, 1059 in zoos and 106 in aquariums.

The number of zoos that were not accredited relative to the number that were differed little among the ten Southeast Asian countries; the only two countries that had lower levels of non-accreditation were Myanmar (zero non-accredited, whereas at least three non-accredited zoos were to be expected) and Singapore (zero non-accredited, whereas we expected at least four) (χ^2^ = 3.31, df = 1, *p* < 0.069 and χ^2^ = 4.14, df = 1, *p* < 0.042, for Myanmar and Singapore, respectively). Combining all the zoos, more accredited zoos offered shows than what could be expected if the number of shows was equally prevalent in accredited and non-accredited zoos (χ^2^ = 7.79, df = 1, *p* < 0.005). Likewise, accredited zoos offered significantly more drive-throughs and more walk- or swim-throughs than would be expected if this was equal between accredited and non-accredited zoos (χ^2^ = 19.36, df = 1, *p* < 0.0001 and χ^2^ = 7.02, df = 1, *p* < 0.008, for drive-throughs and walk- or swim-throughs, respectively). None of the other animal–visitor interactions were significant. Conversely, non-accredited zoos offered significantly fewer drive-throughs (χ^2^ = 4.04, df = 1, *p* < 0.0044). When focusing solely on Thailand, none of these differences was statistically significant. When weighted across countries, there was no statistical difference in the types of animal–visitor interactions in each country, showing that they were prevalent across the countries surveyed.

**Figure 7:**
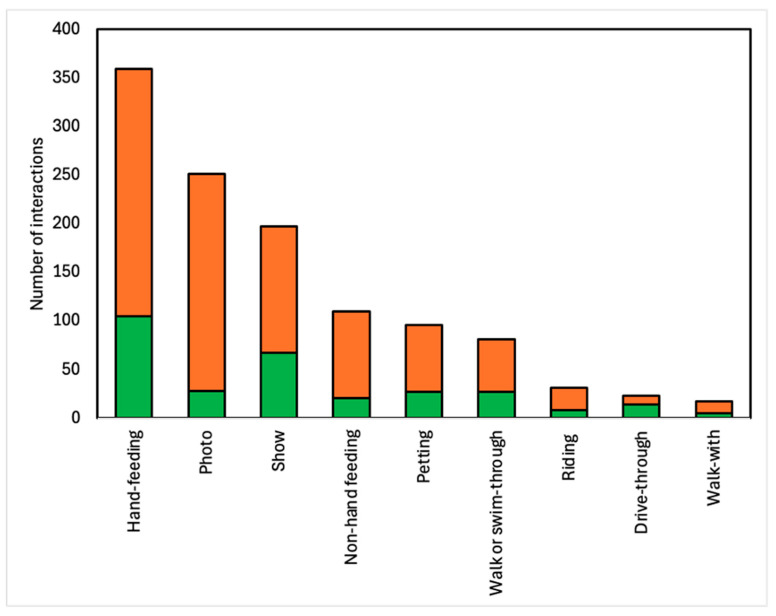
Breakdown of 1165 animal–visitor interactions per type and facility accreditation status. Green represents interactions in accredited zoos, and orange represents interactions in non-accredited zoos.

Of the total 1165 animal–visitor interactions, we found 66% (*n* = 768) were direct interactions where visitors could have physical contact with the animals. Hand-feeding was the most common animal–visitor interaction overall, representing almost one-third of the total interactions, with 31% (*n* = 359) of the total animal–visitor interactions recorded in this study (Figure 7). Bottle-feeding interactions were common, especially with big cats. Photo animal–visitor interactions were the second most frequent, with 251 (22%) of total animal–visitor interactions. We found 34% (*n* = 397) of animal–visitor interactions were classed as indirect (comprising shows, non-hand-feeding, walk- or swim-through, and drive-through interactions). Shows represented the largest indirect animal–visitor interaction category, encompassing 17% (*n* = 197) of total animal–visitor interactions.

In terms of the species class of the animals involved in animal–visitor interactions, we found that mammals were the most represented (*n* = 613), followed by birds (*n* = 304) and reptiles (*n* = 223). The remaining species classes consisted of Actinopterygii (*n* = 11), Arachnida (*n* = 2), Asteroidea (*n* = 2), Cephalopoda (*n* = 1), Chondrichtyhes (*n* = 140, Echinoidea (*n* = 1), Merostomata (*n* = 1), and Scyphoza (*n* = 1). There were 82 animal–visitor interactions that comprised mixed species classes, predominantly animal shows involving mammals and birds, some walk- or swim-through and drive-through animal–visitor interactions.

**Figure 8:**
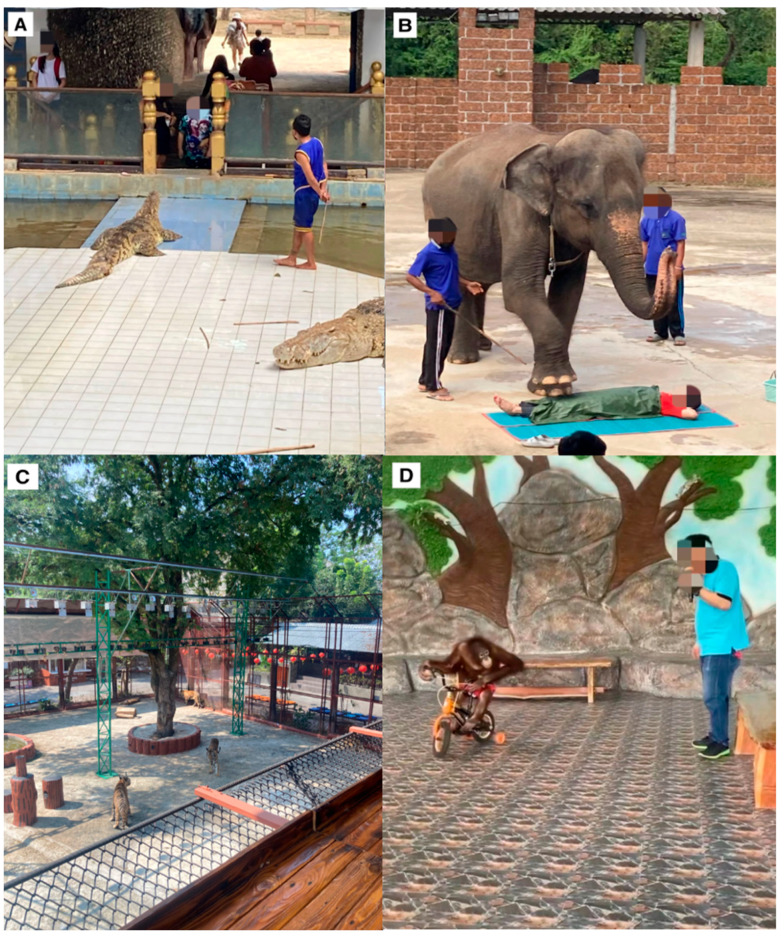
(**A**) Visitors throwing coins inside a crocodile’s mouth; (**B**) elephant walking over a visitor in a show; (**C**) non-hand-feeding interaction whereby visitors are given a rifle to shoot at metal targets containing meat, which, if successful, falls into the enclosure with tigers below; (**D**) orangutan riding a bicycle in a show. Photos were all taken by the first author.

We observed some unusual animal–visitor interactions. Amongst the most uncommon interactions were five incidences of hippopotamus feeding in Indonesian zoos, whereby visitors would enter the enclosure without protection and hand-feed the hippopotamus (*Hippopotamus amphibius*). An animal–visitor interaction in a Filipino zoo involved a visitor entering a transparent plastic box, which was then lowered into a 14-foot saltwater crocodile (*Crocodylus porosus*) enclosure. Another interaction involving crocodiles saw members of the audience in a crocodile show in a Thai zoo throw coins inside a crocodile’s mouth when the trainer solicited tips (Figure 8A). 

We also observed an Asian elephant shows where a visitor would be invited into the arena and lie on the floor. An elephant would hover a foot over the person and walk slowly over the person (Figure 8B). A further unique animal–visitor interaction was a Sunda pangolin (*Manis javanica*) petting in one Indonesian zoo. In aquariums, we recorded ten incidents of petting marine species in touch pools, including sea urchins (Echinoidea), nurse sharks (*Ginglymostoma cirratum*), manta rays (*Mobula alfredi*), horseshoe crabs, and starfish (Asteroidea). During these interactions, visitors subjected animals to continuous handling and touching. One zoo featured an interaction where visitors were given an air rifle and would aim at targets containing meat. If their shot was successful, the meat would drop below into an enclosure with up to nine tigers (Figure 8C). Boxing shows involving Bornean orangutans (*Pongo pygmaeus*) were found in two zoos (one in Thailand and one in Cambodia). Orangutans in the Thai zoo performed various stunts, including zip-lining over the audience or pretending to play instruments; some of the animals were dressed in bikinis and acting as provocative ring girls. Another show featured an orangutan riding a bicycle (Figure 8D). Some animal–visitor interactions did not fit neatly into the assigned categories. For instance, we recorded five incidences of breakfast/dinner with orangutans, giraffes, Asian elephants, and sea lions.

## 4. Discussion

We sought to evaluate the type, prevalence, and species involved in animal–visitor interactions at zoos and aquariums in Southeast Asia. We also investigated whether the zoological association accreditation status impacted the prevalence of animal–visitor interactions. To quantify the impact on animal welfare and conservation messaging, we developed a scoring framework to assess dysfunctionality in animal–visitor interactions, which we used in zoos in Thailand. We found that photos and riding interactions rated most highly for dysfunctionality across accredited and non-accredited zoos and that shows in non-accredited zoos also scored as dysfunctional. We recorded that, at a minimum, 88% of zoos and aquariums in nine of the eleven Southeast Asian countries offered animal–visitor interactions, with no statistical difference in the interaction types across the countries surveyed. The most common animal–visitor interaction type was hand-feeding, followed by photos (including “selfies”) and shows.

### 4.1. Animal–Visitor Interactions’ Prevalence, Geographical Distribution, and Species

As research on the prevalence of animal–visitor interactions in zoos and aquariums is limited, we can only compare our findings to some degree to those of D’Cruze et al. [17]. Their global review only included accredited facilities; therefore, data cannot be compared for non-accredited zoos, which comprised the majority of facilities in our study. The high percentage of animal–visitor interactions found in zoos (96% in Thai zoos and 88% in Southeast Asia overall) was more than the 75% observed by D’Cruze et al. [17]. One reason could be that, as a whole, animal–visitor interactions are more popular amongst visitors to zoos and wildlife tourist attractions in Southeast Asia, who may expect opportunities to have close interactions with captive wildlife during their visit. Such a view is supported by the region’s widespread documented use of animals in entertainment [31,43,47,51,52]. Interestingly, our results found no geographical differences among the animal–visitor interactions offered in the surveyed countries, highlighting that all types of interactions are common throughout the region.

D’Cruze et al.’s [17] finding that petting was the most common animal–visitor interaction amongst global zoos contrasts with our finding that hand-feeding is the most widespread type of interaction in Southeast Asia. Petting represented only a minority of the interactions we found in our online search and in person in Thai zoos. Instead, our findings emphasized the popularity of photos as an animal–visitor interaction, similar to other studies that found taking photographs with a wild animal is a sought-after activity [36,53,54]. The increase in social media usage is undoubtedly a driver as people want to post their photos or “selfies” online [55,56]. Photo opportunities for animal–visitor interactions may exist in some of the globally accredited zoos and were perhaps categorized as petting in D’Cruze et al.’s study [17]. We found that this activity was specifically advertised, therefore warranting its own type of animal–visitor interaction. However, it would be interesting to understand more about this animal–visitor interaction type in accredited zoos worldwide.

As expected, animal–visitor interactions with mammals were most common, as also found by D’Cruze et al. [17], and are consistent generally with mammals forming large components of collection compositions in most zoos [57,58] and being highly popular with visitors [59] Our more in-depth research in Thai zoos indicated that there was not a wide range of species used, with big cats, elephants, crocodiles, pythons, deer, and parrots forming a large component of species. The widespread use of tigers and elephants in Thai zoos corroborates regional and taxa-specific reports highlighting exploitative interactions in zoos and wildlife tourist attractions [42,44,51,60]. As charismatic megafauna, tigers and elephants are some of the most popular species in zoo exhibits worldwide [61]. For this reason, visitors may relish opportunities to be up close and personal to have a photo of the experience to post on social media with such animals [62].

### 4.2. Impact on Animal Welfare

The literature on the welfare impact of animals in animal–visitor interactions is still limited to a few species [11,17]. While there may be some welfare benefits to animals in well-managed interactions [63,64], the scale of dysfunctional animal–visitor interactions uncovered in our research (especially for photos, which had a high dysfunctionality score and which represented the second highest type of animal–visitor interaction) suggests that this is not the case for many animals. Aversive training techniques to force animals to perform unnatural behaviours, such as walking bipedally, using coercion and punishment are well documented [31,65,66,67]. Additionally, some animals are mutilated to become safer to handle, including declawed and de-fanged [36,53]. Furthermore, animals used for some types of interactions may be forced to work all day, depriving them of sufficient sleep and rest [49]. This is even more problematic for nocturnal animals. The physical environment may be poor, as the animal may be exposed to thermal extremes without access to appropriate resources and may be subjected to loud noises, amongst other welfare harms. Moreover, the physical restraint of some animals and the forced proximity between animals and visitors may cause fear and anxiety, especially during petting and photo interactions as animals are touched and held [36,68].

There are many examples of deleterious consequences to welfare caused by certain types of animal–visitor interactions that are too numerous for this discussion. These include animals being given inappropriate food and quantities in hand-feeding or items such as cigarettes, which orangutans in Indonesian zoos have been observed smoking [69]. Animals were also killed when they ingested plastic from food wrappers and other inappropriate items that were thrown into their enclosures [70,71]. Riding elephants causes a multitude of health and psychological problems [43,61,72]. In big cats, the constant demand for small cubs used for hand-feeding, photos, and petting promotes speed breeding as cubs are prematurely removed from their mother to induce her ability to come into heat and have more litters [73]. All in all, the impact on the welfare of the thousands of individual animals involved in animal–visitor interactions is a significant cause for concern and deserves more attention.

### 4.3. Impact on Human Health and Safety

The disregard from facilities for visitor safety was apparent in many animal–visitor interactions we reviewed online and in Thai zoos. Many animal–visitor interactions involve activities with the potential for the visitor to be injured, including fatally so, especially when there is direct contact, such as hand-feeding, petting, photos, and shows. One such example is the elephant walking over a visitor during a show. Over the years, there have been many documented cases in zoos whereby visitors and zoo staff have been injured by captive wild animals, including tigers [74,75,76] and elephants [77,78]. Many tourists appear unaware of the inherent risks involved in animal–visitor interactions [79]. Furthermore, the spread of zoonotic diseases between animals and humans is a danger that is probably unknown to many participating visitors. Primates, for example, are well known to spread hepatitis B and Simian Immunodeficiency Virus, amongst other zoonoses [80,81]. Touching reptiles without washing hands can spread salmonella [82] and tuberculosis can be contracted from ungulates [83,84] and primates [85]. There is also a risk of zooanthroponosis, which is the disease transfer from humans to animals, from animal–visitor interactions, including people passing Influenza A virus methicillin-resistant Staphylococcus aureus to zoo animals [86]. As such, animal–visitor interactions that pose a risk to human health and safety should be terminated. It may be that governments in respective countries are more willing to act on banning dangerous animal–visitor interactions for this reason alone instead of concern for animal welfare.

### 4.4. Impact on Conservation and Education

Positive contributions to conservation and education are core components of modern zoos’ and aquariums’ objectives [87,88] and are increasingly cited by zoos and aquariums to justify their existence [89]. However, there is little evidence that many of the animal–visitor interactions we reviewed contribute to these goals based on the nature of the interactions. Firstly, many people visiting wildlife tourist attractions are not aware of the potentially harmful impact their visit inflicts [18], meaning that visitors themselves cannot be counted on to change their behaviour. Additionally, for visitors who are conscientious enough to show concern as to whether the facility they wish to visit is ethical, the world’s largest travel review site, TripAdvisor [90], continues to approve dysfunctional facilities, stating that these facilities meet their animal welfare policy [91]. Therefore, visitors may continue to engage in dysfunctional animal–visitor interactions because they are unaware of the harm caused. As a result, facilities will continue to offer such interactions as the demand remains. The provision of animal–visitor interactions can cause unethical animal acquisition as some facilities take animals used for entertainment from the wild [92,93]. Wild populations may also suffer as close contact with exotic species, such as in animal cafes, can increase the demand for exotic animals as pets [94] and, in turn, fuel the wildlife trade [55,95,96].

Another worrying aspect of many of the animal–visitor interactions observed in this study is that such activities can negatively influence attitudes and behaviours toward conservation [9]. Many of the interactions feature animals that are being dominated by humans and/or degraded into performing exaggerated, unnatural behaviour, such as the “twerking” observed in elephants and orangutans or primates that are frequently dressed in human clothing. As such, this anthropomorphism only seeks to widen the gulf between humans and nature instead of achieving conservation education’s objective of fostering respect and concern for animals’ habitats [97]. As many visitors to zoos and aquariums are children [98], this issue presents a perturbing matter with lasting multi-generational consequences. Given the urgency of concerns over the increasingly rapid habitat loss and species decline in many areas within Southeast Asia [99,100], the importance of zoos’ and aquariums’ roles in delivering effective conservation education cannot be overstated. However, in many cases, as Walker [26] noted in her paper on dysfunctional zoos, many facilities effectively accomplish the opposite, essentially aiding in the rapid decline of species.

### 4.5. Role of Accreditation

While our finding that less than one-fifth of facilities in Southeast Asia are accredited by SEAZA, the regional zoo association (affiliated with WAZA), may appear extremely low, this is somewhat more than the number of zoos accredited with the Association of Zoos and Aquariums (AZA) in North America, where less than 10% of facilities are accredited [101]. Of course, it may be that, ultimately, the number of accredited zoos in Southeast Asia is comparable since the exact number of zoos in the region is unknown, whereas this is not the case in North America. Nevertheless, one of the questions that needs to be asked here should be about the 44 SEAZA-accredited zoos in our study: Why do not many comply with SEAZA guidelines on animal–visitor interactions? Unfortunately, the answer appears to be that some accredited facilities do not comply, as many animal–visitor interactions are “detrimental to the physical and psychological wellbeing of the animals” [38]. One obvious example is the riding animal–visitor interactions found in accredited zoos.

Yet, these facilities maintain their accreditation status as paying members. Once accreditation has been attained, they may not be subjected to further inspections unless a specific complaint is filed against them. Therefore, as it stands, accreditation may only be of value to a certain extent, as even maintaining the minimal standards required to become accredited does not need to be perpetuated. It is important to note that such an issue is not isolated to SEAZA as a zoological association. In fact, this is an issue even with WAZA members, who may not comply with standards because they are, in effect, just guidelines to achieve the minimum [17,29,102,103]. This issue reminds us that, ultimately, compliance with and commitment to animal welfare, conservation, and education goals are at the will of institution management. For example, there is evidence that accredited zoos in Thailand score significantly better than non-accredited facilities in the provision of signage and quality of animal welfare in hornbills, which, in this case, suggests the value of accreditation [104]. Yet, for many facilities, making a profit may be the priority and trumps all other considerations, hence the continuation of providing dysfunctional animal–visitor interactions. Providing good animal welfare and commercial profitability do not need to be in conflict, as pointed out by Veasey [105], who said that poor animal welfare in Western zoos could lead to that facility’s closure from public outcry alone.

### 4.6. Barriers to Improvement and Recommendations for Change

Understanding why facilities continue to offer dysfunctional animal–visitor interactions may be necessary in order to effectively understand what needs to happen to eliminate such interactions. Barriers to improvement can first be viewed on a societal level, beginning with a society’s understanding and attitudes towards animal welfare, encompassing historical, cultural, and religious elements [106,107,108]. Countries generally implement legislation piecemeal, as and when needed [109]. The Animal Protection Index [110] measures a country’s animal welfare policy and legislation. Currently, the index assesses six Southeast Asian countries (Indonesia, Malaysia, Myanmar, Thailand, the Philippines, and Vietnam), all of which have a low ranking on the index and collectively cover 95% of Southeast Asia’s population [40]. As such, this implies that, in general, animal welfare is not a priority in the region as a whole. 

On an institutional level, as mentioned above, an institution’s organizational objectives essentially control whether profit is prioritized over making positive contributions to conservation, research, animal welfare, education, and providing well-managed entertainment. The issue here is that some facilities may feel conflicted in that they want to act responsibly as zoological institutions but, at the same time, are acutely aware that they must satisfy visitor requirements and expectations for their visit, which often means being suitably entertained [111]. As such, changing people’s attitudes towards engaging in dysfunctional animal–visitor interactions will have to play a large part in creating this behavioural shift. Behaviour change campaigns have been successfully conducted concerning wildlife in Asia, including campaigns on ivory reduction [112,113], shark fin soup [112,114], and bear bile [115]. These campaigns are encouraging as they indicate that, with the help of well-funded animal welfare organizations aligning with influential celebrities, people’s attitudes towards participating in dysfunctional animal–visitor interactions can be changed for the better.

Another important factor often overlooked is examining the conditions for the workers in zoos and aquariums. In many Southeast Asian countries, higher education is not a prerequisite for working as a zookeeper or other roles in a zoo [116]. Coupled with the fact that senior management themselves may not have an educational or professional background in zoos and aquariums [26] makes it more challenging to effectively run a facility that provides good animal welfare and promotes pro-conservation behaviours. Furthermore, compounding these challenges is the lack of the available scientific literature on welfare and husbandry available in languages other than English and the restricted access to such literature, as much data are not open access [103,117]. Finally, with all the best intentions, some facilities operate on meagre budgets and may feel that providing popular but dysfunctional animal–visitor interactions such as photos and rides may be the only way to stay open. However, these facilities may be receptive to receiving assistance, as we will touch upon shortly below.

There are myriad ways to eliminate dysfunctional animal–visitor interactions. As it stands, in addition to working on behaviour change campaigns, legislative measures will have to be initiated since many facilities may be resistant to change [26]. However, for any change to be impactful, various stakeholders (people, animals, biodiversity) must be considered [30], especially for legislation to have the desired effect. Stringent standards must be implemented to control the use of captive wildlife in entertainment, as well as robust zoo standards. At the same time, both SEAZA and WAZA have a duty to ramp up efforts to ensure that existing accredited facilities maintain standards. Once accredited facilities have their own house in order, with a collaborative and committed effort with the zoological association, accredited facilities can help mentor non-accredited zoos to achieve the necessary standards to reach accreditation. A further interesting and potentially powerful tool is for more pressure to be applied to travel organizations such as TripAdvisor to prevent them from displaying facilities that have poor animal welfare (and to ensure that their animal welfare policy that rates facilities is accurate, as currently it is not) [91]. Similarly, in countries such as Thailand, where wildlife tourism forms an important part of the economy [118], it would be beneficial to work with tourism authorities to facilitate an accreditation system that promotes facilities that do not offer dysfunctional animal–visitor interactions.

### 4.7. Limitations and Avenues for Future Research

Our assessment represents a small window into the actual number of animal–visitor interactions available in Southeast Asian zoos and aquariums. There are likely many smaller, non-accredited facilities that receive fewer visitors. As a result, any interactions occurring in these facilities are not being posted online and are consequently unnoticed by us. Furthermore, many smaller facilities may not have a website or social media pages, making it possible for us to miss their facility entirely. What is clear is the wide scope of further research necessary to continue building upon what we know about animal–visitor interactions in Southeast Asia. One area of much-needed research is to talk directly to facilities to understand why they choose to offer dysfunctional animal–visitor interaction and to ascertain why more are not seeking accreditation. Having this information will allow us to build a suitable strategy for increasing accreditation rates by working with SEAZA and accredited facilities to support non-accredited facilities. Another interesting area for future research is to extend our dysfunctionality score assessment to other countries in Southeast Asia and to see how this tool can be used to help facilities themselves self-audit animal–visitor interactions within their facilities. In addition, measuring the impact of dysfunctional animal–visitor interactions on visitor attitudes would be beneficial in creating appropriate behaviour change.

## 5. Conclusions

Our research aimed to understand more about the prevalence and types of animal–visitor interactions in zoos and aquariums in Southeast Asia. It was clear that animal–visitor interactions were found to be widespread in the region, with hand-feeding being the most prevalent. Many animal–visitor interactions in Southeast Asia are dysfunctional, as evidenced by photos being the second most common animal–visitor interaction type and scoring high in our dysfunctionality score. We highlight some of the welfare, conservation, and educational ramifications of such interactions. Despite guidelines from the regional zoological association stipulating that animal–visitor interactions should not cause physical or psychological harm, based on the types of interactions we observed, this was not the case in many incidences. As such, accredited zoos should ensure that they meet association guidelines on animal–visitor interactions and then offer support to help non-accredited facilities improve. Finally, zoos and aquariums are still here to entertain people and, if not regulated, they will allow people to harmfully interact with animals if that provides the required entertainment. In this case, nothing has changed in the last 4500 years in the exploitation of captive wildlife for human amusement. Since animal tourism is predicted to further increase as urbanization increases [119], serious attention to the issues discussed in this study is urgently required.

## Figures and Tables

**Table 1:** Criteria of the different types of animal–visitor interactions as defined by D’Cruze et al., p4 [17], with an additional category of “photo.” A direct animal–visitor interaction refers to interactions whereby the visitor can have direct physical contact with the animal. An indirect interaction is where the visitor is unlikely to expect or receive direct contact with the animal.

Animal–Visitor Interaction	Direct/Indirect	Definition
Petting	Direct	“Interactions where visitors can enter into close proximity with a captive wild animal to hold and touch it, with or without any physical barrier between them, with or without official staff supervision”.
Photo	Direct	Interactions where visitors can enter into close proximity to a captive wild animal, where the interaction is specifically intended for the visitor to have their photograph taken with the animal, whether this photo is taken by facility staff or is a “selfie” with or without a physical barrier between them, with or without official staff supervision.
Hand-feeding	Direct	“Interactions where visitors can enter into close proximity to a captive wild animal and provide food and water by hand, with or without a physical barrier between them, with or without official staff supervision”.
Non-hand-feeding	Indirect	“Interactions where visitors can enter into close proximity with a captive wild animal and provide food and water, although not by hand, with or without a physical barrier between them, with or without official staff supervision. Visitors are likely to have a relatively low expectation of direct contact”.
Riding	Direct	“Interactions where visitors can enter into close proximity with a captive wild animal, which will carry them whilst in motion, with or without a harness or equivalent, with or without official staff supervision”.
Walk or swim-through	Indirect	“Interactions where visitors can experience close proximity to a captive wild animal, which is typically restrained by a harness or equivalent, without any physical barrier, with or without official staff supervision”.
Walk-with	Direct	“Interactions where visitors can experience close proximity to a captive wild animal, which is typically restrained by a harness or equivalent, without any physical barrier, with or without official staff supervision. Visitors are likely to have a relatively moderate expectation of direct contact”.
Drive-through	Indirect	“Interactions where visitors can experience close proximity to a captive wild animal with a vehicle or device acting as a physical barrier, with or without official staff supervision”.
Show and performance	Indirect	“Interactions with trained staff and/or visitors where a captive wild animal provides a demonstration of either natural or non-natural behavior for visitors, with or without a physical barrier between them, under official staff supervision”.

**Table 2:** A scoring framework to assess the dysfunctionality of animal–visitor interactions. The more dysfunctional the animal–visitor interaction, the higher the score (the most dysfunctional is 6, while the least dysfunctional is −6).

Criteria	−1	0	+1
Behavior	Animal performs only natural behaviors.	Animal is not performing unnatural behavior, but the nature of the interaction does not permit an unrestricted behavioral repertoire.	Animal performs unnatural behaviors.
Restraint	Animal has agency as to whether to participate or not in the interaction. No restraint, and has spontaneous and free movement.	Animal is not restrained but cannot remove itself completely from participating or proximity to the interaction.	Animal has no agency whether to participate in the interaction. They are physically restrained (i.e., chained or by staff handling) or in extreme close confinement.
Staff attitude and handling	Confident, patient, gentle, skilled handling, or non-contact, reward-focused training.	Acts indifferently without coercion or intimidation towards the animal.	Domineering, cruel, threatening. Excessive force is used using hand, whip, fist, bamboo, or other tools. Punishment-focused training.
Physical environment	Environmental conditions provide species-specific comfort to the animal.	Environmental conditions may not cause discomfort but do not provide species-specific needs for the animal.	Environmental conditions cause intense physical discomfort to the animal.
Frequency and duration of interaction	The interactions’ frequency and/or duration do not disrupt the animal’s ability to obtain sufficient rest and sleep.	N/A	The interactions’ frequency and/or duration throughout the day likely interrupt the animal’s ability to obtain sufficient rest and sleep.
Conservation messaging	The interaction is conducted to showcase the animal’s natural behavior in a dignified way that can contribute to the visitor’s respect for the animal and appreciation for its need for protection.	Equivocal	A combination of the above factors harms conservation messaging as the interaction does not present the animal as a wild animal deserving of respect and protection.

**Table 4:** Summary of animal–visitor interactions by country, animal–visitor interaction type, and SEAZA accreditation status. The first figure indicates the number of interactions for that interaction type in the country. The figure in bold and parentheses indicates the number of facilities that offer that animal–visitor interaction type. The figure in parentheses underneath represents the percentage of the total number of accredited/non-accredited facilities that offer the animal–visitor interaction type. AVI = animal–visitor interaction.

	Brunei	Cambodia	Indonesia	Laos PDR	Myanmar	Malaysia	Philippines	Singapore	Thailand	Vietnam	Total
Total facilities	2	4	67	2	4	60	22	5	77	12	256
Accredited facilities (% of total)	0 (0)	1 (25)	13 (19)	0 (0)	4 (100)	5 (8)	2 (9)	5 (100)	8 (10)	6 (50)	44 (17)
Facilities with AVIs (%)	1 (50)	3 (75)	58 (87)	0	4 (100)	48 (80)	21 (95)	5 (100)	74 (96)	11	226 (88)
Hand-feeding	4 **(1)** (0/05)	5 **(3)** (100/75)	106 **(42)** (77/59)	0	6 **(4)** (100)	55 **(35)** (80/56)	28 **(16)** (100/70)	9 **(4)** (80)	121 **(52)** (88/64)	24 **(9)** (83/67)	359
Non-hand-feeding	0	0	33 **(24)** (46/33)	0	6 **(2)** (100)	10 **(7)** (20/11)	10 **(6)** (100/20)	0	36 **(24)** (63/27)	1 **(1)** (17/0)	96
Photo	2 **(1)** (0/50)	5 **(1)** (0/33)	88 **(41)** (62/61)	0	1 **(1)** (100)	49 **(24)** (20/42)	28 **(22)** (50/65)	0	71 **(26)** (13/36)	7 **(5)** (17/67)	251
Show	0	9 **(3)** (100/67)	55 **(32)** (77/41)	0	1 **(1)** (100)	29 **(16)** (60/24)	10 **(8)** (50/5)	7 **(5)** (100)	84 **(42)** (100/49)	2 **(2)** (17/17)	197
Petting	1 **(1)** (0/50)	3 **(2)** (100/33)	53 **(24)** (62/30)	0	0	25 **(14)** (0/25)	7 **(5)** (100/15)	0	18 **(15)** (38/17)	3 **(3)** (33/17)	110
Drive-through	0	0	9 **(7)** (23/7)	0	2 **(2)** (100)	2 **(2)** (8/2)	2 **(2)** (50/5)	1 **(1)** (20)	6 **(6)** (25/6)	1 **(1)** (17/0)	23
Walk- or swim-through	1 **(1)** (0/50)	1 **(1)** (100/0)	20 **(16)** (46/19)	0	1 **(1)** (100)	7 **(6)** (40/9)	8 **(7)** (50/30)	2 **(2)** (40)	36 **(33)** (75/38)	5 **(4)** (50/17)	81
Walk-with	0	0	2 **(2)** (8/2)	0	0	2 **(2)** (20/2)	1 **(1)** (0/5)	1 **(1)** (20)	11 **(9)** (25/10)	0	17
Riding	0	0	13 **(13)** (31/17)	0	2 **(2)** 100	2 **(2)** (20/2)	0	0	13 **(13)** (0/19)	1 **(1)** (17/0)	32
Total AVIs	8	23	379	0	20	181	94	20	396	44	1165

Figures in bold and parentheses indicates the number of facilities that offer that animal-visitor interaction type.

## Data Availability

All data are included in the paper; any additional data can be obtained from the corresponding author upon request.
